# Using survival analysis to determine association between maternal pelvis height and antenatal fetal head descent in Ugandan mothers

**DOI:** 10.11604/pamj.2015.22.175.7145

**Published:** 2015-10-22

**Authors:** Ian Guyton Munabi, Samuel Abilemech Luboga, Florence Mirembe

**Affiliations:** 1 Department of Human Anatomy, School of Biomedical Sciences, Makerere University College of Health Sciences, New Mulago Hospital Complex, Kampala, Uganda; 2 Department of Obstetrics and Gynecology, School of Medicine, Makerere University College of Health Sciences, New Mulago Hospital Complex, Kampala, Uganda

**Keywords:** Pelvis height, antenatal diagnosis, childbirth

## Abstract

**Introduction:**

Fetal head descent is used to demonstrate the maternal pelvis capacity to accommodate the fetal head. This is especially important in low resource settings that have high rates of childbirth related maternal deaths and morbidity. This study looked at maternal height and an additional measure, maternal pelvis height, from automotive engineering. The objective of the study was to determine the associations between maternal: height and pelvis height with the rate of fetal head descent in expectant Ugandan mothers.

**Methods:**

This was a cross sectional study on 1265 singleton mothers attending antenatal clinics at five hospitals in various parts of Uganda. In addition to the routine antenatal examination, each mother had their pelvis height recorded following informed consent. Survival analysis was done using STATA 12.

**Results:**

It was found that 27% of mothers had fetal head descent with an incident rate of 0.028 per week after the 25th week of pregnancy. Significant associations were observed between the rate of fetal head descent with: maternal height (Adj Haz ratio 0.93 P < 0.01) and maternal pelvis height (Adj Haz ratio 1.15 P < 0.01).

**Conclusion:**

The significant associations observed between maternal: height and pelvis height with rate of fetal head descent, demonstrate a need for further study of maternal pelvis height as an additional decision support tool for screening mothers in low resource settings.

## Introduction

Maternal body size has been identified as one of the means of estimating successful passage of a fetus through a woman's birth canal. This is based on the observation that in human populations exposed to high disease burden or poor childhood nutrition will tend to have small adult body size proportions in both males and females [1]. This tendency to have small body proportions has also been observed in an ancient Nubian population in which small maternal stature was a risk factor for difficult child birth sometimes ending in death of both mother and fetus [2]. These observations support the link between a combination of childhood malnutrition and infectious disease burden as key factors for development of small maternal stature [3]. Also when these mothers of small body stature, receive improved feeding as adults during pregnancy, they will tend to have a normal sized or even big baby compared to their stature [4, 5]. In the event that the fetal size exceeds any one of the mother's birth canal dimensions, the risk of developing childbirth related injuries for either the mother and baby is markedly increased [1].

Fetal head descent during the antenatal period is currently used as a method for assessing the successful fit and eventual passage of the fetus through the mother's birth canal at the time of childbirth in low resource settings. This assessment is especially important for first time mothers in whom absence of fetal head decent has been associated with a significantly increased risk of complicated childbirth [6, 7]. This risk was further demonstrated by the observation that absence of fetal head descent in 35.8% of deliveries was associated with higher rates of cesarean section birth (P value of 0.042) [8]. Other studies have demonstrated that delayed fetal head descent is a significant risk factor (RR 13.3 CI 3.3-53.0) for terminating labour by cesarean section following arrest disorders [9], low APGAR scores, longer labour and use of augmentation and instruments for delivery [6].

The female birth canal is a highly variable region of the human skeleton [10]. The extent of the observed variability in the size of the birth canal has been associated with active or sedentary life style [11], climatic factors [12] and obstetric requirements [13]. This is because this variability, especially if it leads to a small maternal birth canal, results in increased risk of developing cephalopelvic disproportion [5, 10]. In low resource settings, the high prevalence of malnutrition, acts as an additional biological stressor. This means that a large proportion of mothers in these settings may be at an increased risk of having cephalopelvic disproportion at the time of delivery [10, 14]. This places more emphasis on need to further examine the use of fetal head descent as one of the tools in the planning of childbirth.

It is important to note that in low resource settings like Uganda the malnutrition related increased variability of the maternal birth canal complicates the making of correct clinical judgments especially at the community level health facilities run by low cadre health workers [1, 5, 15]. In more developed settings the use of ultrasound, Ct-scans and Magnetic Resonance Imaging have replaced the more risky pelvimetry radiographs [16–18]. The challenges of: irregular power supply, the cost of these equipment and inappropriateness of some of the measurements with respect to assessment of maternal birth canal dimensions [1] make it important to identify the unique population specific anatomical attributes of the human pelvis to enhance the screening of mothers at risk [19]. In this study we seek for evidence to support the continued use of maternal height and inclusion of maternal pelvis height as additional anthropometric tool for identifying mothers at risk of difficult childbirth in our settings. Such evidence adds credence to the teaching and use of various relatively simple obstetric safety measures which in turn should decrease the risks associated with childbirth locally. In this study we set out to determine the associations between two anthropometric measurements: Maternal height and maternal pelvis height with the rate of fetal head descent in Ugandan mothers during the antenatal period.

## Methods

This was a multi site cross sectional study on 1265 antenatal visit records and examinations of mothers whose key descriptive information is summarized in Table 1. Included in this study were mothers whose gestational age was between 25-40 weeks of pregnancy based on their symphysio-fundal height measurement, made in centimeters by an experienced midwife after obtaining informed consent. During the 14 months of the study starting January 2013 only mothers with a singleton pregnancy were included in the study. These mothers were recruited on each day from each of the participating study site antenatal clinics by a team of previously trained midwives on duty that day. Mothers were recruited from various hospitals in Uganda that included: the National Tertiary Care teaching hospital (Mulago National Referral Hospital), Komamboga health center 4 (in Kampala central Uganda), Kagando hospital (western Uganda), St. Josephs Kitgum hospital (Northern Uganda) and Kilembe hospital (Western Uganda). A summary of the descriptive characteristic of the study population by site is provided in Table 2.

**Table 1:** Descriptive statistics of the study population

Variable	Observations	Mean	SD
Age (years)	1214	24.65	5.29
Height (cm)	1250	158.10	7.15
Height < = 150cm	159	147.23	3.72
Height >150cm	1091	159.69	6.07
Weight (Kg)	1253	62.31	9.84
Fundal height (Cm)	1257	33.61	3.31
Gravida	1254	2.58	2.04
Pelvis Height (cm)	1265	7.63	2.17
Pelvis height < = 7.49cm	690	6.25	0.78
Pelvis height > = 7.50cm	575	9.28	2.16
Head descent	1163	0.27	0.44

**Table 2:** Descriptive statistics for the study population by site

	Site					
Variable	Mulago	Kagando	Kilembe	Kitgum	Komamboga	Total
Height (Mean, (SD))	158.11, (7.33)	154.97, (4.92)	154.46, (5.97)	165.49, (6.41)	159.06, (6.24)	158.10, (7.14)
Pelvis height (Mean, (SD))	9.02, (2.51)	6.72, (0.68)	8.79, (1.09)	7.80, (1.67)	5.26,(0.26)	7.63, (2.17)
**Head descent**						
No	374	271	30	134	45	854
Yes	65	90	6	14	134	309
Total	439	361	36	148	179	1,163

**Figure 1:**
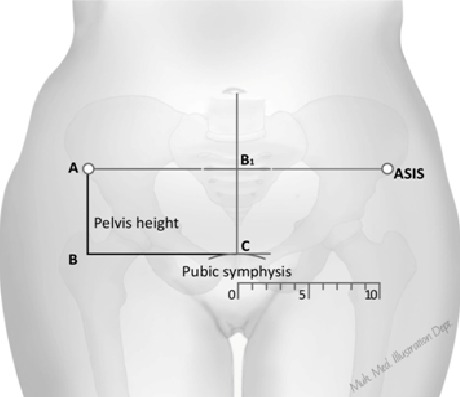
Demonstrating pelvis height using surface landmarks on the female human body

The target sample size was calculated using the sample size calculator for Cox PH regression in STATA 12 to give 1102 mothers, using values from a study pilot for the following input parameters: alpha 0.05, hazard ratio 0.7, power 0.9 withdraws at 70% and expected number of events (fetal head descent) 331. This was inflated by a design effect of 1.15 for the 5 sites to give a total sample size of 1268 participants [20] For each mother, the following information was obtained: Age in years, height in centimeters and weight in kilograms measured using the available hospital equipment [21], gravidity, fetal presentation of the current pregnacy, head descent and symphysio-fundal height in centimeters on clinical examination to the nearest 0.1 centimeter. For each mother the pelvis height in centimeters was measured twice at the time of examination by the attending midwife, using the anterior superior illiac spine (ASIS) and the Symphysis pubis bony body landmarks using a pair of transperent rigid rulers placed at right angles to each other, as demonstrated in Figure 1 (see lines AB and BC). The average of these two measurements was used for analysis. The midwives at each site were trained in how to measure pelvis height and taken through the questionaire at the start of the study with additional refresher trainning and mentorship during the site visits by IGM.

Data was entered into Epidata version 3.2 (Epidata association, Denmark) and exported to STATA 12 (StataCorp LP, Texas, USA) for analysis. The focus of the analysis was on the association between the time to the key endpoint variable was defined as the antenatal visit in which a mother was observed to have fetal head descent on routine clinical obstertic abdominal examination by the research assistant nurse at the given site, with the study anthropometric measurements of maternal height and maternal pelvis height. Survial analysis was used to cater for the time to event nature of the above key end point variable. Also the maternal pelvis height used here is the same as the pelvis height currently used in automotive engineering to delineate the portion of height corresponding to the pelvis in crash test dummies [22]. The maternal pelvis height cut off of 7.50cm [23] was used to generate two groups: the first with pelvis height of less than or equal to 7.49cm and the other group greater than 7.50cm. For maternal height, grouping was done using the traditional cut off value of 150cm to generate two groups: the first with Maternal height of less than or equal to150cm and the other group greater than 150cm. Descriptive statistics were generated using: mean, ANOVA, log rank test, pair wise correlations and Kaplan-Meir survival graph plots for groups generated using the cutoff of 7.50cm [23] for pelvis height. This was then followed by univariable Cox regression modeling. To cater for the study design mulitlevel multivariable discrete time survival analysis using the gllamm function was used to calculate both the harzard ratios. During analysis any record found with a missing value was dropped from analysis and a P <0.05 was considered significant for all tests.

Ethical considerations for this study included obtaining ethical approval from the Makerere University School of Biomedical Sceinces IRB and the study was registered with the Uganda National of Science and Technology. The hospital administrators and heads of units were briefed of the study and the need to obtain a copy of the Antenatal record. All the participating nursing staff were requested verbaly to be part of the study and offered an equivalent of 1US dollar compensation for each Antenatal record filled to completion. For the mothers, each was requried to sign an informed consent form to participate in the study. Informed consent was obtained by the attending midwife for visit. Young mothers less than 18 years, the age of consent in Uganda were handled as emancipated adults and all women were free to consult their spouses or next of kin since the study required one to provide contact information as part of the consent process. With the exception of measuring maternal pelvis height there were no other procedure or modification made to the current routine Antenatal practices at any of the participating sites. Refusal to consent did not result in a mother bieng denied access to health care or required services at the particpating faclity. No identifier marks or personal information was used in the analysis and subsequesnt reporting of the study results.

## Results

**Figure 2:**
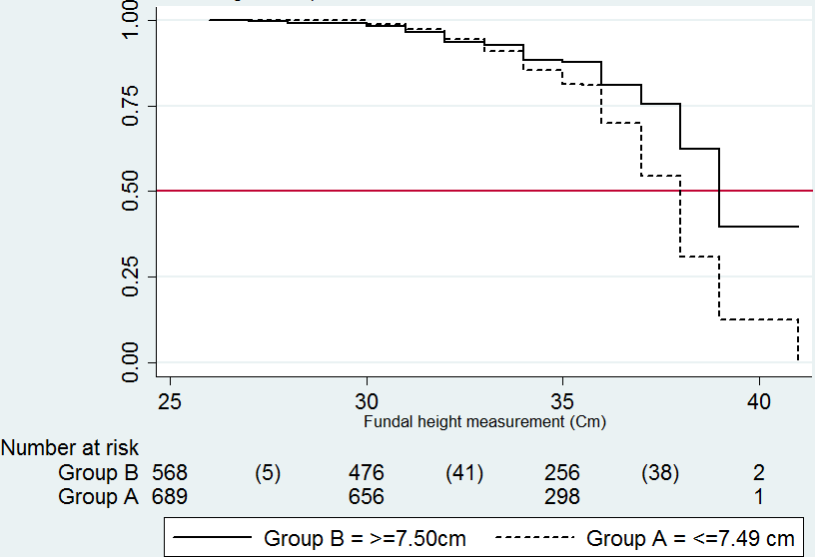
Kaplan-Meier survival estimates for Time of fetal head descent

Out of 1163 mothers, 309 (27%) were found with fetal head descent as summarized in Table 1. The log rank test using the groups made with the maternal height cut off of 150cm, had 276 observations of an expected 284.06 observations for the group of >150cm compared to 33 observations of an expected 24.94 observations for the group < = 150cm. The log rank test for these groups comparisons was not significant (χ^2^= 3.30 P < 0.07). The log rank test for the groups with maternal pelvis height had 225 observations of an expected 187.39 events in the < = 7.49 group compared with 84 observations of an expected 121.61 events in the > = 7.50cm group. The log rank test on this groups comparisons was significant (χ^2^=22.99 P < 0.01). The groups created as a result of categorizing the maternal pelvis height were used to generate a Kaplan-Meir survival graph (Figure 2) which showed the group < = 7.49cm had a higher rate of fetal head descent. The median time to fetal head descent in this group (< = 7.49cm) was 38 weeks compared with 39 weeks in the >= 7.50cm group. There were significant pair wise correlations between: pelvis height and age (-0.09, P = 0.02), maternal height and weight (0.43, P< 0.01) and pelvis height and weight (0.18, P < 0.01). The correlation between maternal height and maternal pelvis height was not significant (0.004, P = 0.89).

The relative risk of observing fetal head descent reduced with increasing maternal height for the groups maternal height <= 150cm compared with maternal height >150cm. This reduction was not significant (RR 0.92, 95% CI 0.67 to 1.26, P = 0.59). The risk of fetal head descent on the other hand increased significantly with increasing maternal pelvis height for the groups maternal pelvis height < = 7.49 compared with maternal pelvis height > = 7.50cm (RR 1.96, 95% CI 1.57 to 2.44, P < 0.01). In Table 3, only maternal pelvis height was found to have a non significant reduction in the risk of fetal head descent on univariable Cox hazard regression.Table 4 provides a summary of the multi variable Cox hazard regression modeling for the different study variables including age, weight and gravidity with respect to time of fetal head descent stratified by the hospital (site) from which the mothers were recruited. In the final model maternal: age, weight and gravidity were not significant and thus removed from the model. The risk of Fetal head descent reduced significantly at a rate of 2% for each unit increase in Maternal height (Adj. Haz. ratio 0.98, P < 0.01) while there was a significant 15% increase in the risk of fetal head descent for each unit increase in maternal pelvis height (Adj. Haz. ratio 1.15, P < 0.01).

**Table 3:** Univariable cox hazard regression models for fetal head descent

Variable	Univariable Haz. Ratio	Standard Error	95% CI (P-value)
Site	1.04	0.01	1.02 to 1.07 (<0.001)
Age	1.04	0.01	1.02 to 1.06 (<0.001)
Height	0.98	0.01	0.97 to 1.00 (0.039)
Weight	0.98	0.01	0.96 to 0.99 (0.002)
Gravida	1.16	0.03	1.10 to 1.22 (<0.001)
Pelvis height	0.93	0.03	0.87 to 1.00 (0.061)

**Table 4:** Multivariate Cox hazard regression model for fetal head descent

Variable	Univariable Haz. Ratio	Adjusted Haz. Ratio^+^	Standard Error	95% CI (P-value)
Age	1.04	0.98	0.01	0.95 to 1.01 (0.17)
Height	0.98	0.98	0.003	**0.97 to 0.98 (<0.01)**
Weight	0.98	1.00	0.01	0.99 to 1.01 (0.85)
Gravida	1.16	1.01	0.04	0.94 to 1.10 (0.74)
Pelvis height	0.93	1.15	0.05	**1.06 to 1.26 (<0.01)**

## Discussion

We set out to determine the associations between two anthropometric measurements: maternal pelvis height and maternal height with the time to fetal head descent in Ugandan mothers during the antenatal period. The data from this study established that both maternal pelvis height and maternal height had significant associations with the time the fetal head descends into the maternal pelvis. Maternal pelvis height was associated with an increased risk of fetal head descent keeping all other factors constant (see Table 4). Also from preliminary observations on preserved rearticulated pelvis bones, pelvis height is strongly correlated with: (1) the mid and outlet dimensions of the birth canal and (2) pelvic inclination [24]. Finally unlike maternal height which reaches a maximum at the age of 16-17 years of age [3, 4], we expect pelvis height to continue changing till the 30th year of life when the growth of the pubis bone ceases in women [14, 25].

Some additional deductions from the above results are: (1), the significant negative correlation between pelvis height and age (-0.09 p value 0.02) supports previous observation that pelvis height reduces with increasing maternal age [26]. (2) The survival analysis and log rank tests in this study allude to an inverse relationship between the rate of fetal head decent and maternal pelvis height. This could be explained by our previously observed positive association between pelvis height and inclination of the birth canal [24]. This means that a mother with a smaller pelvis height will also have a smaller pelvic inclination which places the maternal birth canal inlet in a more horizontal position to receive the descending fetal head. This could be one explanation for the increased rate of fetal head descent in the group with a smaller maternal pelvis height in Figure 2. It is important to note that on multivariable Cox regression this is reversed with the observation of an increased rate of fetal head descent with an increase in pelvis height (Table 4). As demonstrated elsewhere there is a positive association between pelvis height and birth canal dimensions [24]. This means that for any two similar mothers the one with a larger pelvis height would have a correspondingly larger birth canal and thus more space for the descending fetal head in this population. Thus it seems that during the antenatal period maternal pelvis height may influence the rate of fetal head descent through both the position of the birth canal inlet with respect to the descending fetal head and the maternal birth canal dimensions in this population.

Increasing maternal height, was associated with a significant reduction in the rate of fetal head descent in the antenatal period (p value <0.01). This risk rate remained relatively unchanged even with adjustment suggesting that maternal height was a fairly independent factor in prediction of fetal head descent in this population. There are two possible explanations for this: (1) A previously held view based on the rule that a tall woman usually has a big fetus [2, 4], would imply that the bigger/taller the mothers will also have proportionally larger pelvis cavities than the smaller/shorter mothers [2]. This assumption needs to be applied cautiously across diverse populations, since communities characterized by women of small stature may still have adequate pelvis dimensions as observed by more recent research findings by Kurki H. 2011 [1]. (2) The constant nature of the risk associated with maternal height could be explained by the observation that maternal height attains a maximal value at the age of 16-17 years when the growing ends of the long bones fuse [3, 4]. Thus it is expected that throughout the subsequent reproductive years for this mother we would expect the risk to be constant or become non-significant as observed with height and related factors like age and number of pregnancies (Table 4). This historical nature of maternal height as a measure of past maximal bone growth is what makes it a useful indicator of adverse outcomes in settings with chronic malnutrition [14, 27].

One of the short comings of this study was the relatively small sample size for the small hazard ratios under observation. The small sample size was overcome in part using computational simulation strategies to obtain narrower confidence intervals. The other challenge was the nature of the study population which in addition to having low rates of fetal head descent as seen in this study at 27% and 22% from literature [28], also had poor health facility utilization practices [29]. These two observations concerning the study population may further reduce the representativeness of the study findings with respect to the general population. As has been mentioned above fetal head descent is one of the indicators of adequacy of the maternal birth canal. Other measurements used to estimate this adequacy include: maternal height, age and history of previous births [30]. The World Health Organization (WHO) discourages the use of maternal height for example, due to heights’ low power to predict outcomes of pregnancy when used as a diagnostic aid [30, 31]. This study identifies maternal pelvis height, as an additional tool for use in low resource settings for antenatal pelvis assessment.

## Conclusion

This study demonstrates that increasing maternal height is associated with a significant reduction in rate of fetal head descent compared with increasing maternal pelvis height which is associated with a significant increase in rate of fetal head descent in Ugandan mothers during the antenatal period. This is important given that fetal head descent is still used to assess the maternal birth canals capacity to accommodate the descending fetal head at the time of childbirth in low resource settings. There is need for more study to refine the use of maternal pelvis height, which is currently used as pelvis height in automotive engineering, as an additional decision support tool for screening mothers in low resource settings.
